# Association between Participation in Annual Physical Examinations and Risk Factors for Noncommunicable Diseases in Adults with Disabilities: Evidence from Shanghai, China

**DOI:** 10.3390/ijerph17113822

**Published:** 2020-05-28

**Authors:** Yugang Li, Qi Zhang, Xiaohong Li, Mei Sun, Jun Lu, Gang Chen

**Affiliations:** 1 Department of Health Policy and Management, School of Public Health, Fudan University, Shanghai 200032, China; liyugang08@126.com (Y.L.); lixh@fudan.edu.cn (X.L.); sunmei@fudan.edu.cn (M.S.); 2 China Research Center on Disability Issues, Fudan University, Shanghai 200032, China; 3 Key Laboratory of Health Technology Assessment, National Health Commission, Fudan University, Shanghai 200032, China; 4 School of Community and Environmental Health, Old Dominion University, Norfolk, VA 23529, USA; qzhang@odu.edu

**Keywords:** noncommunicable disease, risk factor, annual physical examination, person with disability

## Abstract

The relationships between regular participation in annual physical examinations and the risk factors for noncommunicable diseases (NCDs) among adults with disabilities remains unclear. To address this gap, we used data from 130,899 individuals with disabilities in Shanghai (2014–2016) and evaluated four risk factors for NCDs: hypertension, hyperglycemia, hyperlipidemia, and being overweight. Overall, 4540 individuals participated in annual physical examinations across all three years and 11,388 missed examinations in 2015 (group without regular participation). Chi-squared tests and binary logistic regression were used to assess differences in patient characteristics and explore correlations between variables. Significant differences in age (*χ*^2^ = 102.620, *p* < 0.01), place of residence (*χ*^2^ = 94.308, *p* < 0.01), educational level (*χ*^2^ = 59.884, *p* < 0.01), marital status (*χ*^2^ = 16.414, *p* < 0.01) and disability type (*χ*^2^ = 56.499, *p* < 0.01) and severity (*χ*^2^ = 45.464, *p* < 0.01) were found between those who participated in regular physical examinations and those who did not. Regular participation was associated with reduced incidences of hypertension (odds ratio 0.799, 95% confidence interval (CI): 0.733–0.871) and hyperlipidemia (0.347, 95% CI: 0.307–0.392), but not with the incidence of diabetes (1.049, 95% CI: 0.944–1.166) or being overweight (0.907, 95% CI: 0.812–1.014). Hence, regular participation in annual physical examinations had different associations with risk factors for NCDs.

## 1. Introduction

The health of people with disabilities has attracted worldwide attention [1]. The World Health Organization (WHO) and the World Bank jointly released the first World Disability Report on 9 June 2011 [2]. The report provided the most comprehensive collection of data on disabilities and the findings created a scientific basis for implementing the United Nations Convention on the Rights of Persons with Disabilities. In 2014, the 67th World Health Assembly adopted the WHO Global Disability Action Plan for 2014–2021, the theme of which was to improve health for all persons with disabilities [3]. This plan treats disabilities as a global public health issue and focuses on improving the health, function, and well-being of persons with disabilities.

The World Disability Report shows that more than one billion people worldwide (approximately 15.6% of the global population) have some type of disability [4] and >85 million of them are in China (China Disabled Persons’ Federation, 2018). In China, chronic diseases have become the most common health threat [5], accounting for >80% of deaths each year, which is equivalent to about 10.3 million deaths [6]. Noncommunicable diseases (NCDs), such as uncontrolled hypertension, elevated fasting blood glucose levels, hyperlipidemia and being overweight, coexist with disability and increase the disease burden among people with disabilities [7]. Due to mental, physical, and structural impairments or disorders, those with disabilities face a more serious risk of chronic disease than other people [8].

Research showed that few countries have effective and appropriate measures for the management and prevention of chronic diseases. However, in controlling the risk factors of chronic diseases, many countries have adopted a series of health management measures and strategies, including health checkups, to prevent the development and worsening of chronic diseases. Previous studies largely used cross-sectional health examination data obtained from people with disabilities to analyze differences associated with different disability types, sex, and other sociodemographic characteristics, as well as to examine the effect of health-related indicators among people with disabilities. However, research is lacking on the association of annual physical examinations and the risk factors for chronic diseases in people with disabilities.

We aimed to investigate the association between regular participation in annual physical examinations and the risk factors for NCDs in adults with disabilities. Shanghai is the most developed city in China with a population of about 2.4197 million (2016), of which approximately 512,863 people have one or more disabilities. To investigate the association between regular health examinations and NCD risk factors among those with disabilities, we used annual health examination data collected over three years (2014, 2015, and 2016) in Shanghai, China. We divided the population according to whether they participated in annual physical examinations across all three years. The purpose of our study was to compare and analyze the association of continuous participation in annual physical examinations with risk factors for NCDs in persons with disabilities. We found that continuous annual physical examinations are associated with certain risk factors for NCDs, but not with others. 

## 2. Materials and Methods

Since 2004, Shanghai has provided free annual medical examination services for persons with disabilities. The Shanghai Disabled Persons’ Rehabilitation Comprehensive Information Platform (SHDPRCIP) was established by the Shanghai Disabled Persons’ Federation to collect health examinations and sociodemographic data from persons with disabilities in Shanghai. The inclusion of participants into the registry is voluntary but requires them to undergo a professional medical and functional assessment by a qualified physician based on the International Classification of Functioning, Disability, and Health guidelines. The type and severity of the disability are identified and classified after the assessment. The overall examination for people with disabilities includes physical, imaging, and laboratory examinations. Physical examinations include basic measurements (height, weight, blood pressure, etc.) and organ examinations (heart, lung, liver, spleen, anus, eyes, ears, nose, throat, etc.), whereas imaging examinations include abdominal ultrasonography (liver, gallbladder, spleen, and pancreas), electrocardiography and chest radiography. Laboratory tests include routine blood tests, blood biochemical tests, routine urine tests, and immunology tests. During the examination, doctors in each department first collect basic data. A chief doctor then analyzes all the information, including the laboratory results, and provides a final report for every person with a disability.

In 2014, 2015, and 2016, a total of 46,108, 41,431, and 43,360 people in Shanghai participated in the annual medical examinations, respectively. We collected physical examination data from people with disabilities in Shanghai over this three-year period and divided the population into two groups according to whether they participated in the annual physical examinations in all three years. We hypothesized that those who participated in the annual physical examinations would be more likely to have risk factors for certain chronic NCDs than would those who did not. We selected four important risk factors for chronic NCDs: hypertension, hyperglycemia, hyperlipidemia and being overweight. We explored the relationship between annual physical examination participation and the risk factors for these NCDs.

This study was approved by the Institutional Review Board (IRB) of the Fudan University School of Public Health (IRB number 2015-08-0563). All subjects provided written informed consent before participating in the health examinations. Only adults aged >18 years were included in this study.

### 2.1. Independent Variable

Participation in annual health examinations for three consecutive years (2014–2016) was considered as the independent variable in the study. A total of 4540 individuals with disabilities in Shanghai participated in the annual physical examinations in 2014, 2015, and 2016. A total of 11,388 people with disabilities missed the annual check in 2015 and were included in the group without regular participation in the annual physical examinations.

### 2.2. Dependent Variables

Hypertension, hyperglycemia, hyperlipidemia, and being overweight were considered as dependent variables. Hypertension was defined as a systolic blood pressure of ≥140 mmHg, a diastolic blood pressure of >90 mmHg, or both, based on the revised 2011 Chinese Guidelines for the Prevention and Treatment of Hypertension [9]. Hyperglycemia was defined as a fasting blood glucose level of ≥6.1 mmol/L according to the 2013 Chinese Guidelines for the Prevention and Treatment of Diabetes (China Diabetes Federation, 2014) [10]. Hyperlipidemia was defined as a total cholesterol level of ≥5.2 mmol/L or triglyceride level of ≥1.7 mmol/L according to the 2016 Chinese Guidelines for the Management of Dyslipidemia in Adults [9]. Overweight was defined as a body mass index (BMI) of ≥24 kg/m^2^ according to the recommended BMI for the Chinese population [11].

### 2.3. Covariates

This study included two sets of covariates. The first set consisted of sociodemographic characteristics including sex, age group, place of residence, educational level, and marital status. The place of residence was categorized as either urban or rural based on the participant’s current address. The age groups were 18–29, 30–39, 40–49, 50–59, 60–69, and ≥70 years. The educational level was classified as elementary school or lower, middle school, and high school or higher. Marital status was classified as never married, married, divorced, or widowed. The second set of covariates included the type and severity of the disability. The professional medical institutions designated by the Shanghai Disabled Persons’ Federation conducted the disability assessment according to the Chinese National Standard for the Classification and Grading Criteria of Disability (GB/T 26341-2010) [12]. The results of the assessment (i.e., the type and severity of the disability) were registered in the Health Promotion Plan. The type of disability was categorized as hearing, speech, visual, physical, intellectual, mental, or multiple disabilities [11]. Bodily functions affected by the type of disability were scored and the severity of disability was divided into four levels on the basis of the Chinese National Standard for the Classification and Grading Criteria of Disability (China, 2011). Levels one and four corresponded to the least and most severe levels of the disability, respectively. Cases of multiple disabilities involved the presence of two or more disabilities and were graded according to the criteria for the most severe disability.

### 2.4. Statistical Analysis

Descriptive statistics were calculated and qualitative data are expressed as numbers and percentages. Pearson’s chi-squared test was used for comparisons between groups. Logistic regression analysis was used to explore differences in health outcomes according to regular participation in annual health examinations after adjusting for covariates. The correlation coefficient for the independent variable and covariates was <0.5, indicating no significant multicollinearity. Individuals who did not participate in examinations across all three years were considered the reference group; odds ratios (ORs) and 95% confidence intervals (CIs) were estimated for each risk factor in the other group. A *p*-value of < 0.05 was considered statistically significant. Data analysis was performed using SPSS Statistics version 22.0 (IBM, Armonk, NY, USA).

## 3. Results

Table 1 shows the sociodemographic and disability characteristics of the two groups in our study: those who participated in the annual physical examinations regularly and those who did not. 

There were 8674 men, accounting for 54.46% of participants, and slightly fewer women (7254, 45.54%). There was no significant difference in age between the two groups (*χ*^2^ = 2.937, *p* = 0.087).

The 60–69 years age group was the most represented (38.99%). Of those who participated in annual physical examinations regularly, the most highly represented age group was the 50–59 years group (35.37%). The 60–69 years age group was the predominant group among those who did not participate annually (40.52%). The age difference between the two groups (annual and non-annual participants) was statistically significant (*χ*^2^ = 102.620, *p* < 0.01).

More than half of the persons with disabilities in this survey had urban residence permits (82.41%). Among those who participated annually, the proportion of those with an urban residence permit (87.05%) was higher than that of those with a rural residence permit (12.95%). The proportion of non-annual participants with urban residence permits (80.56%) was also higher than the proportion with rural residence permits (19.44%) and the difference in the type of residence permits between the two groups (annual and non-annual participants) was significant (*χ*^2^ = 94.308, *p* < 0.01).

More than half of the survey respondents had at least a middle-school education (78.07%). Among the persons with disabilities who participated in physical examinations annually, those with a middle school education accounted for the highest proportion (51.30%). Similarly, even among those who did not participate annually, those with a middle-school education were the predominant group (54.21%). The educational level of those who did not participate annually was higher than that of annual participants. This difference between the two groups was statistically significant (*χ*^2^ = 59.884, *p* < 0.01).

Most study participants were married (79.77%). Among those who participated in the physical examination annually, most were married (78.37%) and this was also true for non-annual participants, for whom the proportion of married persons was 80.32%. The difference in marital status between the two groups was statistically significant (*χ*^2^ = 16.414, *p* < 0.01).

Physical disabilities accounted for the highest percentage of disabilities (48.92%). The proportion of those with a physical disability among annual participants was 49.65%. Among non-annual participants, the proportion of persons with a physical disability was 48.63%. The types of disability showed statistically significant differences between the groups (*χ*^2^ = 56.499, *p* < 0.01).

A level four disability was the most common in the study population (45.79%). This was true both among annual and non-annual participants (42.16% and 47.24%, respectively). The disability level difference between the two groups was statistically significant (*χ*^2^ = 45.464, *p* < 0.01).

The analysis of risk factors for NCDs is shown in Table 2.

**Figure 1:**
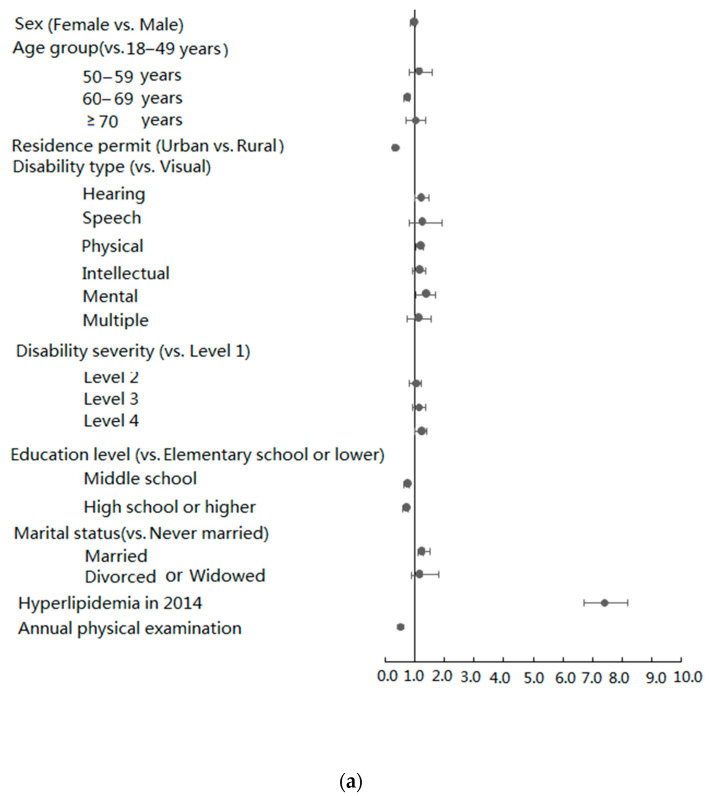

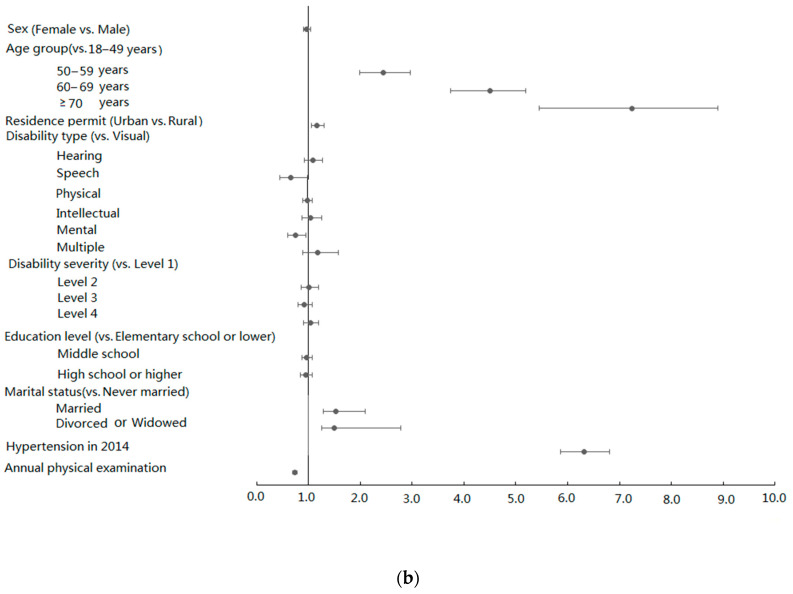
Analysis of risk factors for (**a**) hypertension (**b**) and hyperlipidemia.

The results showed that the risk of hypertension increased with age. However, speech impairment, mental disability, and annual physical examination were protective factors for hypertension and their ORs (95% CIs) were 0.644 (0.434–0.956), 0.759 (0.601–0.958), and 0.799 (0.733–0.871), respectively. The occurrence of hypertension among participants was significantly different in the year 2014 (OR 6.280, 95% CI 5.823–6.772). The risk factors associated with hyperlipidemia, specifically in 2014, included hearing impairment (OR 1.231, 95% CI 1.016–1.490), mental disability (OR 1.437, 95% CI 1.124–1.837), level four disability (OR 1.206, 95% CI 1.010–1.441), and hyperlipidemia (OR 7.351, 95% CI 6.659–8.114). A higher education level was associated with a lower risk of hyperlipidemia (middle school: OR 0.680, 95% CI 0.606–0.763; high school or higher: OR 0.638, 95% CI 0.554–0.734). Annual physical examination was also a protective factor for hyperlipidemia (OR 0.347, 95% CI 0.307–0.392). Conversely, the relationship between annual participation in physical examinations and diabetes or overweight showed ORs (95% CIs) of 1.049 (0.944–1.166) and 0.907 (0.812–1.014), respectively. In general, the prevalence of hypertension and hyperlipidemia was related to annual participation in the physical examinations, whereas that of hyperglycemia and overweight was not (Figure 1).

## 4. Discussion

### 4.1. Demographic Characteristics

The survey data used in this study were provided by the Shanghai Disabled Persons’ Federation and consisted of the annual health checkup data of people with disabilities in Shanghai. Baseline data showed no difference in the proportion of men between participants who underwent physical examinations annually and those who did not. Among people aged >60 years, the proportion of annual participants was lower than that of non-annual participants (35.18% vs. 40.52%). This difference may be due to reduced mobility associated with older age and an already high number of hospital visits. Notably, the educational level of non-annual participants was higher than that of annual participants. This may be attributed to people with a higher educational level usually belonging to a higher social class and, as it is the work unit that manages and implements the annual physical examinations, people with a higher educational level may be less willing to participate [13].

### 4.2. Hypertension

A previous study showed that regular participation in annual physical examinations is an effective method to prevent disease and promote health [14]. At present, the most effective method for controlling hypertension is early detection, which enables early prevention and the implementation of control measures [15]. Compared with people without disabilities, people with disabilities are at a higher risk of hypertension [16], which may be related to their less healthy lifestyles [17]. In our study, participating in annual physical examinations was found to be a protective factor against hypertension after the adjustment for other confounding factors. Annual physical examination can help the timely discovery and early management of risk factors for hypertension [18].

### 4.3. Hyperlipidemia

Chen et al. conducted a survey of people with disabilities living in the Zhabei area of Shanghai in 2008. The results showed that the prevalence of hyperlipidemia was highest (17.1%) among people with chronic diseases and this condition was more prevalent than hypertension (15.3%) and diabetes (6.0%) [19]. Thus, the prevalence of hyperlipidemia in people with disabilities cannot be ignored.

We found that hearing impairments and mental disabilities were risk factors for hyperlipidemia. People with hearing impairments and mental disabilities are less likely to acquire knowledge and skills to practice appropriate lifestyle habits [20], putting them at an increased risk of developing hyperlipidemia. In contrast, a higher educational level translates to more attention toward maintaining good physical health and thus a lower risk of developing hyperlipidemia and other diseases [21]. In this study, after adjusting for other confounding factors, participating in annual physical examinations was found to be a protective factor against hyperlipidemia. Hence, annual physical examinations can help in the early detection and management of hyperlipidemia.

### 4.4. Hyperglycemia

The prevalence of diabetes has increased in recent years, which may be related to the increasing prevalence of unhealthy lifestyles [22]. However, the results of this study showed that annual participation in physical examinations was not directly related to diabetes, possibly because most patients with diabetes take medication regularly to control blood glucose levels.

### 4.5. BMI

A previous study has shown that overweight is a risk factor for diabetes and hypertension [16]. Hove [23] reported that younger people with disabilities are more likely to be overweight or obese. The reason for this may be related to the need for people with disabilities to take medication because of their disability, leading to obesity. Another reason could be that people with disabilities are less able to perform physical exercise, making it challenging for them to maintain a healthy weight [24]. These findings indicate that maintaining a normal weight is particularly important for people with disabilities. In this study, there was no correlation between participation in annual physical examinations and overweight. This may be due to the generally high BMI among people with disabilities and the lack of health education regarding weight control during physical examinations. Long-term follow-up studies are still required to clarify and confirm these findings.

### 4.6. Limitations

Although this study specifically included people with disabilities in Shanghai, there were some limitations in the sampling. Collecting data on other risk factors of chronic diseases, such as genetic, environmental and behavioral factors, was difficult. We did not find that diabetes and overweight were associated with annual participation in physical examinations. The occurrence and development of chronic NCDs is a long-term process; thus, long-term research is needed. Lastly, at the time of blood sampling, we did not consider medications taken for hypertension, hyperlipidemia, and diabetes, and not doing so may have led to an underestimation of the chronic disease rate to some extent.

## 5. Conclusions

This study revealed that regular participation in annual physical examinations had different associations with risk factors for NCDs. In particular, annual physical examinations were associated with a lower incidence of hypertension and hyperlipidemia but were not associated with that of diabetes and overweight. Therefore, it is necessary to individualize health examination programs and measures for people with disabilities to improve the efficiency and value of health examinations. Our research provides reliable supporting evidence for formulating more scientific and effective physical examination policies for people with disabilities.

## Figures and Tables

**Table 1:** Sociodemographic and disability characteristics of the participants by physical examination group.

Characteristic	Total		Annual Group ^1^		Non-Annual Group ^2^		*χ* ^2^	*p*
	*n*	%	*n*	%	*n*	%		
Sex								
Male	8674	54.46	2521	55.53	6153	54.03	2.937	0.087
Female	7254	45.54	2019	44.47	5235	45.97		
Age group, years								
18–49	2786	17.49	964	21.23	1822	16.00	102.620	<0.01
50–59	5331	33.47	1606	35.37	3725	32.71		
60–69	6211	38.99	1597	35.18	4614	40.52		
≥70	1600	10.05	373	8.22	1227	10.77		
Place of residence								
Urban	13,126	82.41	3952	87.05	9174	80.56	94.308	<0.01
Rural	2802	17.59	588	12.95	2214	19.44		
Educational level								
Elementary school or lower	3493	21.93	1175	25.88	2318	20.35	59.884	<0.01
Middle school	8503	53.38	2329	51.30	6174	54.21		
High school or higher	3932	24.69	1036	22.82	2896	25.44		
Marital status								
Never married	2242	14.08	718	15.81	1524	13.38	16.414	<0.01
Married	12,705	79.77	3558	78.37	9147	80.32		
Divorced or Widowed	981	6.15	264	5.82	717	6.30		
Type of disability								
Visual	4102	25.75	1084	23.88	3018	26.5	56.499	<0.01
Hearing	1162	7.3	304	6.70	858	7.53		
Speech	174	1.09	24	0.53	150	1.32		
Physical	7792	48.92	2254	49.65	5538	48.63		
Intellectual	1696	10.65	531	11.70	1165	10.23		
Mental	704	4.42	252	5.55	452	3.97		
Multiple	298	1.87	91	2.00	207	1.82		
Severity of disability								
Level 1	1546	9.71	466	10.26	1080	9.48	45.464	<0.01
Level 2	2353	14.77	778	17.14	1575	13.83		
Level 3	4735	29.73	1382	30.44	3353	29.44		
Level 4	7294	45.79	1914	42.16	5380	47.24		

^1^ Annual group: participated in the physical examinations annually (all three years); ^2^ non-annual group: did not participate in the physical examinations annually.

**Table 2:** Analysis of risk factors for chronic noncommunicable diseases.

Variable	OR ^1^ (95% CI)			
	Hypertension	Hyperlipidemia	Diabetes	Overweight
Sex (female vs. male)	0.966 (0.895–1.043)	0.951 (0.868–1.042)	1.088 (0.987–1.199)	0.904 (0.817–1.000)
Age group, years (vs. 18–49)				
50–59	2.383 (1.953–2.908)	1.047 (0.841–1.303)	0.865 (0.733–1.021)	0.922 (0.754–1.126)
60–69	4.346 (3.587–5.265)	0.720 (0.574–0.904)	0.984 (0.840–1.152)	0.996 (0.819–1.210)
≥70	7.142 (5.609–9.092)	0.729 (0.526–1.012)	1.084 (0.876–1.342)	1.007 (0.765–1.325)
Place of residence (urban vs. rural)	1.202 (1.081–1.337)	0.345 (0.309–0.384)	0.879 (0.771–1.003)	1.093 (0.953–1.254)
Type of disability (vs. visual)				
Hearing	1.080 (0.923–1.263)	1.231 (1.016–1.490)	0.877 (0.715–1.075)	1.033 (0.836–1.277)
Speech	0.644 (0.434–0.956)	1.128 (0.739–1.722)	1.056 (0.663–1.683)	0.904 (0.552–1.480)
Physical	0.982 (0.894–1.078)	1.212 (1.078–1.363)	0.918 (0.813–1.037)	0.967 (0.851–1.098)
Intellectual	1.039 (0.869–1.242)	1.094 (0.899–1.330)	0.939 (0.761–1.159)	1.250 (1.004–1.558)
Mental	0.759 (0.601–0.958)	1.437 (1.124–1.837)	0.879 (0.673–1.147)	1.492 (1.136–1.961)
Multiple	1.185 (0.888–1.581)	1.059 (0.729–1.540)	0.921 (0.642–1.323)	1.113 (0.763–1.624)
Severity of disability (vs. level 1)				
Level 2	1.017 (0.866–1.194)	1.039 (0.849–1.271)	1.022 (0.835–1.251)	1.070 (0.866–1.321)
Level 3	0.924 (0.794–1.076)	1.167 (0.967–1.407)	1.035 (0.856–1.252)	1.055 (0.864–1.288)
Level 4	1.037 (0.900–1.196)	1.206 (1.010–1.441)	1.011 (0.845–1.209)	1.146 (0.950–1.384)
Educational level (vs. elementary school or lower)				
Middle school	0.954 (0.862–1.056)	0.680 (0.606–0.763)	0.925 (0.815–1.050)	1.148 (1.005–1.311)
High school or higher	0.926 (0.821–1.044)	0.638 (0.554–0.734)	0.974 (0.838–1.133)	1.088 (0.929–1.274)
Marital status (vs. never married)				
Married	1.763 (1.481–2.100)	1.357 (1.056–1.743)	1.218 (1.024–1.448)	0.874 (0.723–1.058)
Divorced or Widowed	1.664 (1.260–2.918)	1.404 (0.949–2.078)	1.171 (0.886–1.549)	0.784 (0.563–1.091)
Chronic diseases in 2014 *	6.280 (5.823–6.772)	7.351 (6.659–8.114)	24.527 (22.059–27.271)	64.415 (58.297–71.175)
Annual physical examinations	0.799 (0.733–0.871)	0.347 (0.307–0.392)	1.049 (0.944–1.166)	0.907 (0.812–1.014)

* This variable was adjusted regardless of the occurrence of two or more of the chronic noncommunicable diseases in 2014. ^1^ Odd Ratio.
